# Supplementary material for: Zearalenone Promotes Uterine Development of Weaned Gilts by Interfering with Serum Hormones and Up-Regulating Expression of Estrogen and Progesterone Receptors
Source: Toxins (Basel). 2022 Oct 26;14(11):732. doi: 10.3390/toxins14110732 (PMC9695532; doi:10.3390/toxins14110732)
Supplement: Supplementary file 1 [file toxins-14-00732-s001.zip › toxins-1947616-supplementary.pdf]

toxins

MDPI

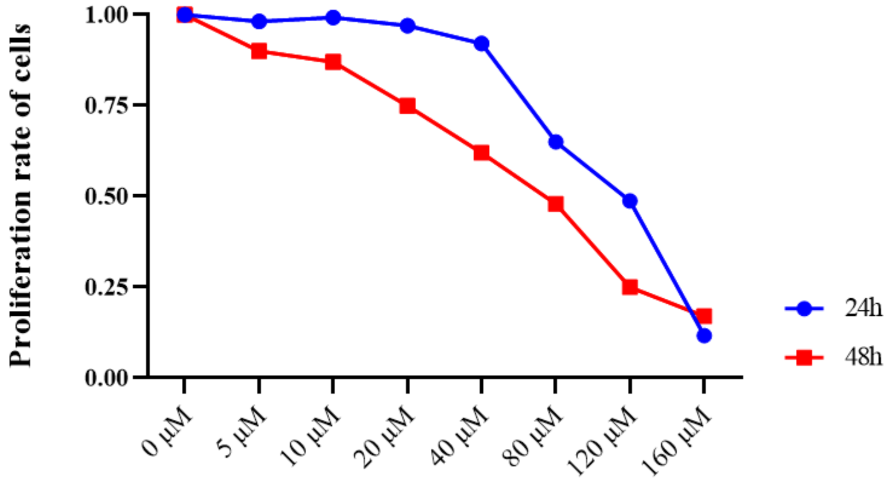


**Figure S1.** Effects of zearalenone on cell viability of porcine endometrial epithelial cells, exposed to ZEA at 0, 5, 10, 20, 40, 80, 120 and 160 μmol/L for 24 h and 48 h.

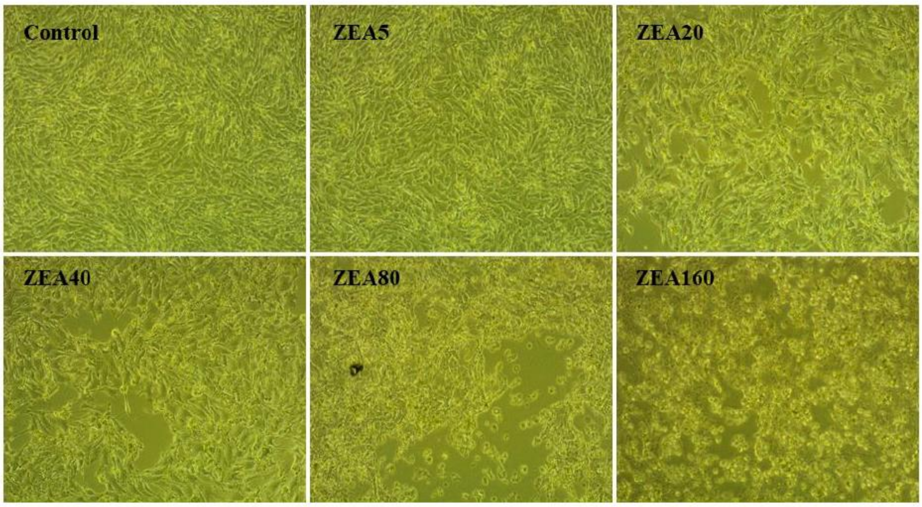


**Figure S2.** Effects of zearalenone on the morphology of porcine endometrial epithelial cells, exposed to ZEA at 0, 5, 20, 40, 80 and 160 μmol/L for 24 h.
